# COC α DA - a fast and scalable algorithm for interatomic contact detection in proteins using C α distance matrices

**DOI:** 10.3389/fbinf.2025.1630078

**Published:** 2025-09-01

**Authors:** Rafael Pereira Lemos, Diego Mariano, Sabrina De Azevedo Silveira, Raquel C. de Melo-Minardi

**Affiliations:** 1 Laboratory of Bioinformatics and Systems, Department of Computer Science, Federal University of Minas Gerais, Belo Horizonte, Brazil; 2 Laboratory of Bioinformatics, Visualization and Systems, Department of Informatics, Federal University of Viçosa, Viçosa, Brazil

**Keywords:** COCαDA, protein interactions, contacts, structural bioinformatics, command-line tool

## Abstract

Protein interatomic contacts, defined by spatial proximity and physicochemical complementarity at atomic resolution, are fundamental to characterizing molecular interactions and bonding. Methods for calculating contacts are generally categorized as cutoff-dependent, which rely on Euclidean distances, or cutoff-independent, which utilize Delaunay and Voronoi tessellations. While cutoff-dependent methods are recognized for their simplicity, completeness, and reliability, traditional implementations remain computationally expensive, posing significant scalability challenges in the current Big Data era of bioinformatics. Here, we introduce COC α DA (COntact search pruning by C α Distance Analysis), a Python-based command-line tool for improving search pruning in large-scale interatomic protein contact analysis using alpha-carbon (C α ) distance matrices. COC α DA detects intra- and inter-chain contacts, and classifies them into seven different types: hydrogen and disulfide bonds; hydrophobic effects; attractive, repulsive, and salt-bridge interactions; and aromatic stackings. To evaluate our tool, we compared it with three traditional approaches in the literature: all-against-all atom distance calculation (“brute-force”), static C α distance cutoff (SC), and Biopython’s NeighborSearch class (NS). COC α DA demonstrated superior performance compared to the other methods, achieving on average 6x faster computation times than advanced data structures like *k*-d trees from NS, in addition to being simpler to implement and fully customizable. The presented tool facilitates exploratory and large-scale analyses of interatomic contacts in proteins in a simple and efficient manner, also enabling the integration of results with other tools and pipelines. The COC α DA tool is freely available at https://github.com/LBS-UFMG/COCaDA.

## 1 Introduction

Proteins are essential biological macromolecules, composed of amino acid residues linked by covalent peptide bonds. Their final three-dimensional structure is shaped not only by these covalent connections but also by weaker interactions such as hydrogen bonds, electrostatic forces, and hydrophobic effects (Smetana and Misra, 2017). The correct folding and stability of proteins are critical for their biological functions, making structural analysis fundamental for understanding cellular mechanisms, identifying therapeutic targets, and guiding the development of new drugs.

Since the first experimental resolution of a protein structure in 1958 (Kendrew et al., 1958), the field of structural biology has seen tremendous advances. Initiatives such as the Protein Data Bank (PDB, (Berman et al., 2000)) and, more recently, the AlphaFold Protein Structure Database (AFDB, (Varadi et al., 2024)) have centralized experimentally resolved and computationally predicted protein structures, making them widely accessible. The rapid growth of these repositories reflects not only advances in experimental techniques, such as X-ray crystallography, NMR spectroscopy, and cryo-electron microscopy, but also the impact of computational modeling approaches, including deep learning-based tools such as AlphaFold2 (Jumper et al., 2021) and AlphaFold3 (Abramson et al., 2024). These advances are part of the “Big Data era in Bioinformatics”, characterized by challenges related to data storage, processing, and interpretation at scale (Mura et al., 2018; Pal et al., 2020; Mariano et al., 2023).

The PDB currently holds 238,922 entries, with approximately 92% corresponding to protein structures^1^. The archive continues to grow at an annual rate of around 6.5%^2^, driven by both experimental and computational contributions (Kovalevskiy et al., 2024). This exponential expansion highlights the urgent need for computational strategies that can efficiently organize, validate, and analyze structural data at large scale. In particular, it is crucial to develop tools capable of supporting fundamental research, as well as applications in biomedical and biotechnological fields.

One key aspect of protein structure analysis is the characterization of interatomic contacts. Contacts are defined as spatial relationships between atoms or residues either within a molecule or between molecules, and are crucial for understanding protein-protein interactions, structural stability, and ligand binding mechanisms (da Silveira et al., 2009; Pires et al., 2011). In this context, it is important to distinguish between “contacts”, defined purely by spatial proximity, and “interactions”, which imply energetic contributions such as hydrophobic or electrostatic forces (Godzik et al., 1992; da Silveira et al., 2009). While not every contact results in a functional interaction, the presence of contacts is often a prerequisite for biologically relevant interactions. Therefore, in the remainder of this paper, the terms contact and interaction may be used interchangeably where appropriate, with “contact” referring primarily to spatial proximity and “interaction” to biochemical context.

Computational methods for contact identification offer an efficient alternative to labor-intensive experimental approaches, facilitating large-scale analyses across protein families and databases (Ding and Kihara, 2018). Traditionally, contacts are identified using Euclidean distance thresholds or cutoff-independent methods such as Voronoi (Voronoi, 1908) or Delaunay tessellations (Delaunay, 1934). Although cutoff-independent approaches are more sophisticated in theory, distance-based methods are often preferred for their simplicity, efficiency, and interpretability (da Silveira et al., 2009; Pires et al., 2011). Recent refinements incorporate physicochemical characteristics such as polarity or charge alongside spatial proximity, improving the biological relevance of computational predictions and reducing the incidence of false positives.

Several tools and databases have been developed to identify and analyze protein contacts (Wallace et al., 1995; Mancini et al., 2004; Schreyer and Blundell, 2009; Laskowski and Swindells, 2011; Bickerton et al., 2011; Pires et al., 2011; Schreyer and Blundell, 2013; Jubb et al., 2017; Fassio et al., 2020; Pimentel et al., 2021). However, existing solutions often present one or more limitations: they may be static, based on predefined datasets; computationally expensive, hindering large-scale use; restricted by server bottlenecks; limited to specific contact types such as residue-residue or protein-ligand; based on cutoff-independent methods; unsupported for modern file formats such as mmCIF; or discontinued altogether.

While these algorithms are well-established in the literature and typically can perform well for single structures, their computational cost becomes a bottleneck in large-scale analyses. Although our current study is based on experimentally determined structures from the PDB, the underlying method is designed with scalability in mind. The landscape of available protein structures has been further expanded by ultra-large-scale prediction initiatives. Notably, the AFDB now provides access to millions of high-confidence predicted models, vastly increasing the volume of structural data available for analysis. This shift underscores the growing importance of methods that combine accuracy with computational efficiency, as the feasibility of analyzing such extensive datasets hinges on scalable algorithms. In addition, time-resolved techniques such as molecular dynamics (MD) simulations introduce another dimension of complexity. These simulations generate thousands of frames per trajectory, each representing a unique protein conformation. Performing contact calculations across such datasets requires algorithms that can process structural information repeatedly and efficiently.

In response to these challenges, we propose COC α DA (COntact search pruning by C α Distance Analysis), a novel, Python-based approach for efficient large-scale identification of inter- and intrachain atomic contacts in proteins. COC α DA applies optimized contact cutoffs derived from a systematic analysis of all protein structures in the PDB, leveraging maximum C α distances to enhance accuracy and consistency. The tool features a customized parser capable of handling both PDB and mmCIF formats, offering options for large file management, residue and contact filtering, and geometric property calculations such as centroids and normal vectors for aromatic residues. To support scalability and flexibility, COC α DA allows parallel batch processing across multiple CPU cores, and user-defined custom contact distances.

To validate and benchmark COC α DA, we performed two case studies: a small-scale benchmark involving gold-standard enzyme superfamilies to compare against existing slower methods, and a large-scale application covering all PDB entries with fewer than 10,000 residues. These evaluations demonstrate COC α DA’s capacity for accurate, high-throughput structural analysis, opening new avenues for research in protein evolution, pathogen mutation tracking, virtual compound screening, and beyond. COC α DA can also be easily adapted to any existing analysis workflow, or be run independently for exploratory purposes.

## 2 Methodology

**FIGURE 1:**
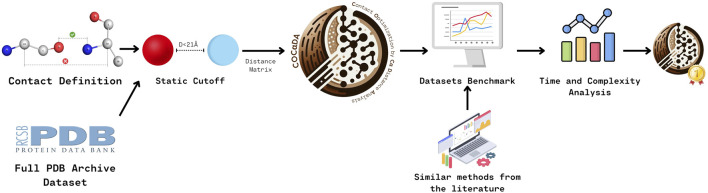
Overview of the methodology used to create and benchmark COC α DA. Initial contact definitions were based on previously published studies. Along with a general implementation of contact detection using fixed cutoff distances, these conditions were applied to the full set of protein structures available in the PDB. This first step led to the creation of a distance matrix, resulting in the improved implementation called COC α DA. COC α DA was then compared to different methods available in the literature using two distinct datasets. Finally, the results were analyzed in terms of processing time and complexity, demonstrating that our tool outperforms its competitors.

Figure 1 outlines the methodology for developing and benchmarking COC α DA. The process begins with defining contacts and applying a static cutoff distance to the full PDB dataset. COC α DA then uses the maximum possible C α distance matrix to improve contact detection. The tool was benchmarked against similar methods using two datasets, focusing on processing time and computational complexity.

### 2.1 Contact definition

To store the contact types and their conditions, we used a dictionary containing all heavy atoms from the 20 standard amino acids, as defined in (Sobolev et al., 1999; Silva et al., 2019; Fassio et al., 2020; Barroso et al., 2020; Pimentel et al., 2021; Dos Santos et al., 2022). All 20 standard amino acids had their heavy atoms classified by the following characteristics, in binary form (Table 1): tendency to contribute to hydrophobic effects, belonging to aromatic groups, having positive charge, having negative charge, capability of donating or accepting electrons. The full atom classification table is available in the Supplementary Table S1.

**TABLE 1:** Example of the binary classification of heavy atoms, according to their characteristics. For each amino acid residue, all their heavy atoms were classified in a binary manner, according to the following characteristics (hydrophobic, aromatic, positive, negative, donor, acceptor). Atom names follow the PDB nomenclature.

Residue	Atom	Hydrophobic	Aromatic	Positive	Negative	Donor	Acceptor
Alanine	N	0	0	0	0	1	0
Arginine	NH2	0	0	1	0	1	0
Glutamate	OD1	0	0	0	1	0	1
Glycine	CA	0	0	0	0	0	0
Tryptophan	CZ2	1	1	0	0	0	0

The possible contact types are: hydrogen and disulfide bonds; hydrophobic effects; attractive, repulsive, and salt bridge interactions; and aromatic stackings. This dictionary also contains the conditions needed for the contact (e.g., to form an attractive interaction, the atoms must be differently charged), and the range of Euclidean distances, in angstroms, for the contact to occur (Table 2).

**TABLE 2:** Summary of Types, Range and Conditions for contacts to occur. ${\text{D}}_{a}$ = Euclidean distance between the atom pair.

Contact type	Range (Å)	Condition (other than range)
Hydrogen Bond	$0\leq D_{\text{a}}\leq 3.9$	Acceptor + Donor atoms
Disulfide Bond	$0\leq D_{\text{a}}\leq 2.8$	Cys:SG + Cys:SG atoms
Hydrophobic	$2.0\leq D_{\text{a}}\leq 4.5$	Hydrophobic + Hydrophobic atoms
Repulsive	$2.0\leq D_{\text{a}}\leq 6.0$	Equally charged atoms
Attractive	$3.9\leq D_{\text{a}}\leq 6.0$	Differently charged atoms
Salt Bridge	$0\leq D_{\text{a}}\leq 3.9$	Equally charged atoms + hydrogen bonding
Aromatic Stacking	$2.0\leq D_{\text{a}}\leq 5.0$	Centroids of two aromatic rings in parallel or perpendicular orientation

### 2.2 Protein Data Bank archive

The full PDB protein archive, in ‘.cif’ format, was obtained using in-house scripts to query and download entries directly from the PDB API. First, a script was used to query the API for entries containing “Protein” as an exact match from the parameter “entity_poly.rcsb_entity_polymer_type”.

To avoid rate limits and overwhelming the server, queries had a 1 s delay from one another, and only 25,000 IDs were obtained at a time. Then, a second script was used, together with the Biopython Bio. PDB module (Cock et al., 2009), to download all files that matched the IDs gathered in the first step. All files were downloaded between July 4th and 10 July 2024.

### 2.3 Neighbor search implementation using biopython

To serve as a comparison to our method, the Biopython package (Cock et al., 2009), largely used in bioinformatics, was used. The Bio. PDB module contains tools to parse a. pdb or. cif file, as well as the NeighborSearch (NS) class, which is useful in interatomic contact determination.

We used an in-house Python script to perform an all-atom neighbor search of 6Å radius, the maximum distance for contacts defined in our dictionary. Then, the neighbors were filtered based on their distance and physicochemical properties relative to the parent atom. Redundant comparisons were excluded; for example, if atom “ a ” was identified as a neighbor of atom “ b ,” the comparison was performed only once, preventing the redundant evaluation of “ b ” as a neighbor of “ a .” The code for the NS implementation is available at the Supplementary GitHub repository.

The contacts obtained contained the following information: chain, residue number, and parent atom name; chain, residue number, and neighbor atom name (i.e., the atomic pair making the contact); type of contact; and distance between the two atoms.

### 2.4 General implementation

To analyze the PDB protein archive and obtain the maximum distances matrix used in the rest of this work, we first devised a Static Cutoff (SC) implementation, where the C α cutoff distance was fixed. Akin to Biopython, proteins are treated as Python objects, containing chains, residues, and atoms. The package includes a customized. pdb/.cif parser, optimized to rapidly extract only the information relevant for contact determination. This makes it more efficient and lightweight than general-purpose parsers, which are typically designed to support a broader range of structural analysis tasks. By default, the parser considers the following criteria: a) Only the first model of each protein is considered (in the case of proteins experimentally resolved by NMR); b) Only atoms with occupancy ≥ 0.50 are considered; c) Water molecules, hydrogen atoms, non-standard residues, nucleic acids (DNA and RNA), and metallic coordination are not considered.

After parsing, the protein object is passed to a contact calculation script, where the C α distances for each pair of residues are obtained, and filtered based on the fixed cutoff. Centroids of aromatic rings were calculated using all atoms belonging to the ring, and the calculation of normal vectors and angles was performed using the Python NumPy library.

The atoms from the residues that are in range to interact are then compared to the dictionary previously described, based on their distance to each other, and their physicochemical properties. Finally, the contacts are returned in a custom object containing all their information, similar to the NS method.

### 2.5 Distance matrix

Throughout the processing of the complete PDB archive using the SC method, the maximum distances (across all proteins in the PDB) between the C α atoms of each amino acid pair were stored in a distance matrix. Upon completion of the processing, this distance matrix was then used to update the static cutoff point employed in the SC method, generating specific values for each amino acid pair.

The distance matrix $D={\left[d_{ij}\right]}_{n\times n}$ is a square matrix of size n×n , where n represents the number of standard amino acids. Each entry $d_{ij}$ corresponds to the maximum distance between the C α atoms of the amino acids at positions i and j (e.g., $d_{11}$ represents an Alanine pair, and $d_{nn}$ represents a Valine pair):

$$D=\left[\begin{array}{cccc} d_{11} & d_{12} & \cdots & d_{1n}\\ d_{21} & d_{22} & \cdots & d_{2n}\\ \vdots & \vdots & \ddots & \vdots \\ d_{n1} & d_{n2} & \cdots & d_{nn} \end{array}\right],$$ (1)

where each $d_{ij}$ represents the maximum Euclidean distance between the C α atoms of the amino acids at positions i and j .

A total of 210 distance values were obtained, representing each possible residue pair and excluding redundancies (e.g., Ala-Val is the same as Val-Ala) (Equation 2).

$$P=\frac{n\left(n-1\right)}{2}+n,$$ (2)

where P is the number of non-redundant distance pairs, and n is the number of standard amino acids. In this case, as n = 20, then P = 210.

### 2.6 COC α DA implementation

**FIGURE 2:**
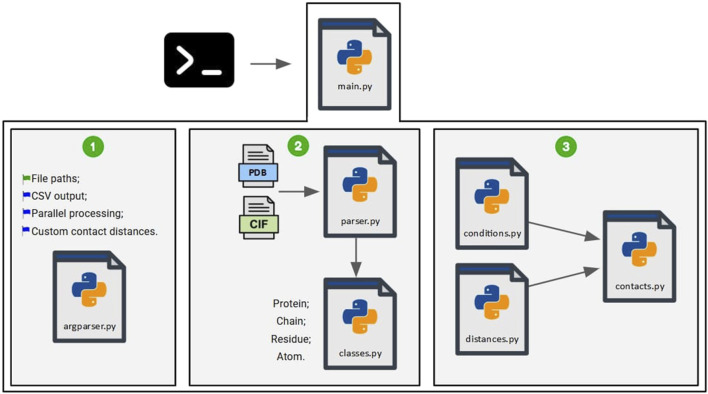
Scripts used for the COC α DA implementation. The steps for processing the command-line parameters (1), processing the input files and creating the objects (2), and calculating contacts using the values obtained from the distance matrix (3) are shown.

The COC α DA tool is implemented similarly to the previous implementations (NS and SC). A schematic representation of the implementation is presented in Figure 2.

First, the script “main.py” is executed via the command line, along with the required parameters, which are processed by the script “argparser.py” (1). The parameters include the mandatory file paths (wildcards are accepted), an optional binary flag for generating an output file in. csv format (default = no), an optional parameter to parallelize file processing in batches across any combination of available CPU cores, and an optional parameter to use custom contact distances defined by the user instead of those defined in Table 2 (using the “contact_distances.json” configuration file). All parameters are fully explained using the ‘-h’ or ‘–help’ flags.

When users specify custom contact cutoffs greater than the default (6Å), the static C α –C α distance matrix is extended accordingly. This is done by computing an epsilon (ϵ) value, which is the difference between the user-defined cutoff and the default maximum cutoff. If ϵ>0 , it is added to all distance values in the original matrix. This adjustment ensures that the pruning based on C α distances remains valid even under relaxed contact definitions, preserving consistency with the originally computed matrix while accommodating user-defined thresholds.

The files are then processed by the script “parser.py” (2), which utilizes the previously defined classes to create objects representing proteins, their chains, residues, and atoms. Finally, the script “contacts.py” receives the objects generated by the parser (3). The scripts “conditions.py”, which stores the conditions required for contact detection, and “distances.py”, which stores the values obtained from the distance matrix, are used to compute the contacts.

If the user does not specify the. csv output file parameter, only a summary is displayed in the terminal, containing the protein name, residue count, number of contacts, and processing time in seconds. If the parameter is used, in addition to the summary, a. csv file is generated with detailed information about each detected protein contact. The columns in the output file are organized as follows: Chain 1, Residue Number 1, Residue Name 1, Atom Name 1, Chain 2, Residue Number 2, Residue Name 2, Atom Name 2, Distance, Contact Type. An example output file for PDB ID 101M, as well as a PyMOL (Schrödinger, LLC) visualization script to help users quickly explore the results, are available at the Supplementary GitHub repository.

### 2.7 Datasets

Two datasets were selected to benchmark our results and compare them to other competitors ( $n_{\mathrm{dataset1}}$ = 896 and $n_{\mathrm{dataset2}}$ = 215,716). The first (D1) is a modified gold-standard set of enzyme superfamilies (Brown et al., 2006), with 365 unique entries ranging from 194 to 6,208 residues. For a more balanced comparison, we split all chains in different files, and treated them separately. The new modified dataset contains 896 entries, ranging from one to 994 residues (available on the Supplementary GitHub repository). The second dataset (D2) includes all PDB proteins with less than 10,000 residues, covering approximately 99.2% of all protein entries. This dataset contains 215,716 unique entries, ranging from three to 10,000 residues.

### 2.8 Benchmarks

To ensure fairness and eliminate biases, all benchmarks were conducted simultaneously on a server with the following specifications: NVIDIA A100 GPU, 768 GB RAM, and a 128-thread AMD Ryzen Threadripper 5995WX processor. To prevent memory overload and parallelization issues, each process was executed on an individual core. Due to its size, dataset D2 was divided into nine batches of approximately 25,000 files each, with each batch being processed independently on separate cores.

Although multithreading and batch processing is available for all implementations (NS, SC, and COC α DA) using the Python module “concurrent.futures”, each core handled only a distinct batch to maintain consistency in the results. The total processing time for each entry was defined as the sum of the file reading, parsing, contact detection, and output generation times.

## 3 Results and discussion

### 3.1 Maximum distance matrix

In total, 217,454 PDB entries were downloaded in. cif format, totaling approximately 450 GB (The full ID list is available on the Supplementary GitHub repository). Proteins ranged from three (PDB IDs: 1Q7O, 8DDG, 8DDH) to 503,221 (PDB ID: 8GLV) modeled amino acid residues. To obtain the values for the distance matrix, we processed all the downloaded files using a fixed C α distance cutoff of 21Å for all pairs of residues (SC). This value is comfortably above the maximum distance between the C α of a pair of arginines, the biggest residues by length, that are able to have contacts between their side-chain atoms (considering a maximum contact distance of 6Å, as per Table 2). To confirm this, we compared an all-atom approach (i.e., comparing every atom of the protein against each other, without cutoffs) to the SC approach using D1, and no contacts were missed (Supplementary Table S2).

**FIGURE 3:**
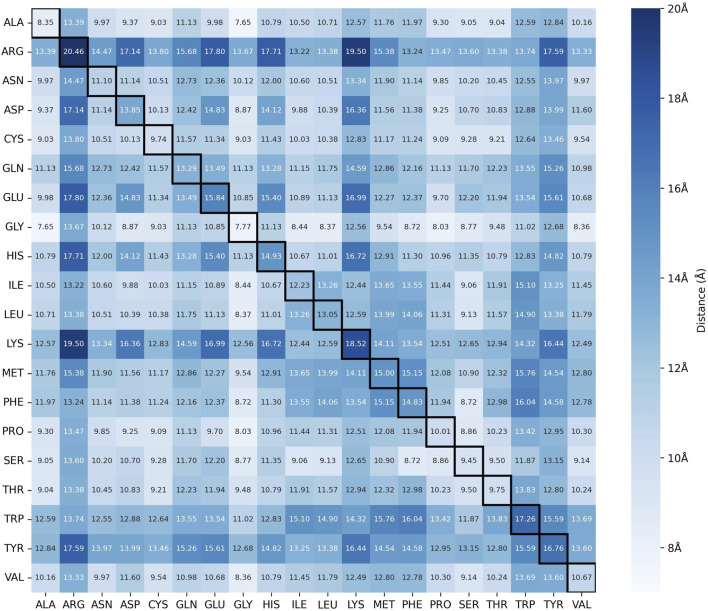
Distance Matrix between the C α of all pairs of residues. The intensity of the color indicates the scale of the value (from 7 to 20 Å). The highlighted diagonal represents pairs of the same residue (e.g., Ala-Ala). The full list of values is available in the Supplementary Table S3.

Using the SC implementation and the 217,454 entries downloaded from the PDB, over 211 million amino acid residues and 819 million contacts were processed and identified. Along this process, we stored the maximum C α distances for every pair of the 20 standard amino acids, and after merging redundancies, we obtained 210 values in a symmetric distance matrix (Figure 3; Equation 1). The full distance table is available in the Supplementary Table S3.

**FIGURE 4:**
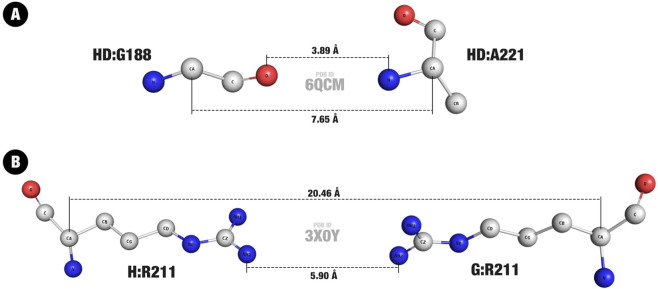
Minimum and maximum entries in the Distance Matrix. **(A)** Minimum value, in a hydrogen bond between a glycine and an alanine residue. **(B)** Maximum value, in a repulsive interaction between two arginine residues. The higher number (larger dotted line) represents the distance between C α , and the lower one (smaller dotted line) the contact distance. The PDB IDs are shown in center, and the contact details are shown in the format Chain:Residue-Atom.

As the distance matrix is color-coded based on the value of the maximum C α distance, we can quickly spot the minimum and maximum values obtained. For the lowest value encountered, we found a pair consisting of an alanine and a glycine residue, both present in the HD chain of PDB ID 6QCM, with a distance of 7.65Å between their C α ’s (Figure 4A). This is expected, as alanines and glycines are two of the smallest amino acid residues, differing only by a single ${\text{CH}}_{3}$ group in the side chain of the alanine, while glycine has a hydrogen atom in its side chain. However, even with this difference, the presence of the ${\text{CH}}_{3}$ group on the side chain of the alanine does not impact the distance between their C α atoms, only contributing to the chirality of the alanine residue. For these two residues, only hydrogen bonds are possible, as the main chain atoms are only capable of donating (main chain nitrogen) or accepting (main chain oxygen) hydrogen atoms.

On the other hand, a pair of two arginine residues, from chains G and H of PDB ID 3X0Y, represents the highest C α distance encountered, of 20.46Å (Figure 4B). This result is also expected and consistent with the fixed distance of 21Å used in the SC approach, once again demonstrating that the fixed cutoff was appropriate to yield no missed contacts. The contact itself is a repulsive interaction between two ${\text{NH}}_{2}$ atoms from the residues side-chains, at a distance of 5.9Å.

If the user wishes to apply custom contact distances instead of those defined in Table 2, the optional ‘-d’ flag can be used, specifying the desired values in the “contact_distances.json” configuration file. This flag extends the C α distance values in the distance matrix to incorporate the user-defined distances, ensuring that no contacts are omitted.

Use-case scenarios for modifying the default cutoff distances include adopting alternative minimum or maximum values reported in the literature, such as 6Å for aromatic stacking (Fassio et al., 2022), or 5Å for hydrophobic effects (Bickerton et al., 2011), instead of the default 5Å and 4.5Å, respectively. Another common scenario involves exploratory analyses using step-wise cutoff variations (da Silveira et al., 2009; Vangone and Bonvin, 2015).

### 3.2 COC α DA

With the maximum possible C α distances properly established for all amino acid residue pairs, we updated with the new values the “distances” dictionary from the SC implementation (Section 2.4), which before was fixed at 21Å for all residue pairs. To the joint implementation of the SC method with the distances updated from the distance matrix, we gave the name COC α DA.

By using tightly-defined, pair-specific cutoff distances, we can further enhance the search space pruning compared to using a single fixed distance threshold. Analysis of the distribution of maximum C α distances reveals that most amino acid pairs exhibit distances well below 21Å, suggesting that introducing pairwise-specific cutoffs is justified and efficient (Supplementary Figures S1 and S2). Moreover, given that dictionary lookups in Python operate with linear average-case complexity (O(1)), storing and querying 210 unique cutoff values introduces negligible computational overhead relative to using a single fixed value.

To improve efficiency, COC α DA employs a stepwise filtering strategy that first evaluates residue-level proximity based on C α –C α distances. An initial coarse filter excludes residue pairs exceeding the global maximum cutoff (20.46Å, for Arg–Arg pairs), allowing early removal of clearly non-interacting pairs. Remaining pairs are then subjected to a more stringent, pair-specific cutoff comparison using the distance matrix. Only those pairs that satisfy both criteria proceed to atomic-level evaluation to determine whether a contact is present. This tiered approach reduces the number of atomic comparisons required, helping to balance computational cost with contact detection accuracy. A schematic illustration of this process is provided in the Supplementary Figure S3.

The residue-pair-specific distance matrix used in COC α DA captures the full range of C α –C α distances observed across known protein structures that are compatible with side-chain atomic contacts. Because these thresholds are grounded in the physical constraints that govern residue-residue contacts, the method is broadly applicable regardless of the overall fold, resolution, or origin of the structural model. Any contact that can realistically occur must still conform to these spatial constraints, ensuring generalization across diverse structural contexts.

While the method is robust to structural variability at the backbone level, the accuracy of contact detection may be influenced by uncertainty in side-chain atom positions, particularly in lower-resolution or flexible regions. In such cases, performance near cutoff boundaries may be affected, which is an inherent limitation of any deterministic cutoff-dependent method.

### 3.3 Benchmarks

To benchmark COC α DA against other approaches used in the literature, we selected the following: all atoms against all atoms (AllAtoms, used in (Pimentel et al., 2021)), Arpeggio (Jubb et al., 2017), Arpeggio CLI^3^ (Jubb et al., 2017), Biopython Neighbor Search (NS, used in (Bickerton et al., 2011)), and Static Cutoff (SC). Other methods, like nAPOLI (Fassio et al., 2020), STING Contacts (Mancini et al., 2004), and PICCOLO (Bickerton et al., 2011), were not available at the time of search and were not updated recently, so they were not considered.

Both Arpeggio versions were too slow to process even small proteins, as our tests showed processing times of approximately 5 and 23 min for a single 1,000 residue protein (PDB ID 6RTH) for Arpeggio CLI and Arpeggio Web, respectively. For comparison, the same protein was processed in 0.62s using COC α DA. This can be due to several factors, but we believe that the explanation lies mainly in server load (for Arpeggio Web), and the several external libraries and computing time that are needed to run the more complex analysis (for both versions). In this way, since a large-scale analysis would not be feasible due to the large processing times, both versions (Web and CLI) were disregarded in the subsequent analyses.

For the AllAtoms approach, a new implementation was developed in Python, in order to incorporate all the previously defined definitions and constraints, and for the NS method, a custom implementation was created using Biopython (Cock et al., 2009), since the PICCOLO tool is currently unavailable. Thus, for D1, the following methods were compared: AllAtoms, NS, SC and COC α DA.

The first dataset contains 896 entries, ranging from one to 994 residues. The initial choice of a smaller dataset was made to include the AllAtoms approach, which is significantly slower than the other three (but still considerably faster than both versions of Arpeggio), which would make a large-scale analysis unfeasible.

**FIGURE 5:**
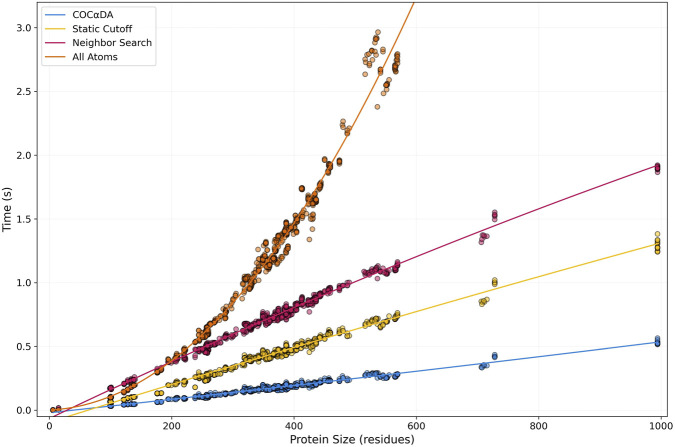
Protein Size vs. Computation Time plot of Benchmark 1. In the first benchmark, 896 files ranging from 1 to 994 residues were analyzed. COC α DA is shown in cyan, SC in yellow, NS in magenta, and AllAtoms in orange. Points represent individual entries, with lines showing the fitted curves for the data.

In Figure 5, it is possible to see that the AllAtoms approach (orange) rapidly explodes in a quadratic curve compared to the three others, which maintain rather linear calculation times up to 1,000 residues. Once again, no contacts of any type were missed in any of the approaches (Supplementary Table S2), but the AllAtoms approach was removed from further analysis because of its performance. Comparing the faster approaches, SC (yellow) obtained calculation times 1.5x faster on average than NS (magenta), while COC α DA (cyan) showed calculation times 3.8x faster on average, obtaining the same contacts.

As the results from the first, small dataset showed a significant difference in processing times between the 3 fastest approaches, we then moved to D2, which contains 215,716 unique entries, ranging from three to 10,000 residues, making approximately 99.2% of the PDB protein archive. The choice to remove entries above 10,000 residues was made due to the nature of those entries, which are mostly protein complexes, containing several copies of each unique chain. This makes them not suitable for contact analysis directly, requiring some kind of pre-processing, like splitting only the unique chains or working with each individual protein present in the complex separately. This can also be true for entries below 10,000 residues, but we believe that this slice correctly represents the diversity of experimentally resolved protein structures.

**FIGURE 6:**
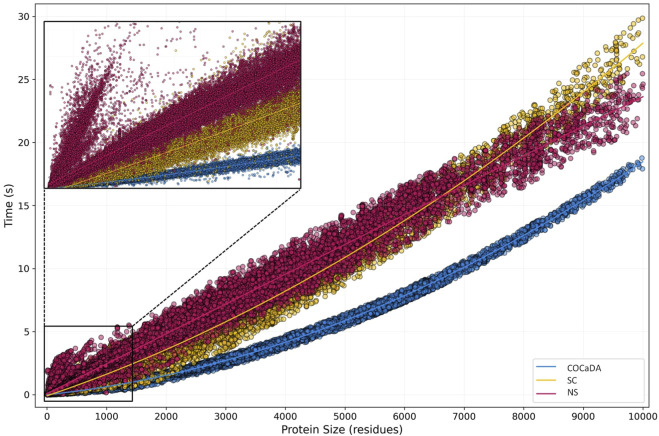
Protein Size vs. Computation Time plot of Benchmark 2. In the second benchmark, 215,716 files were analyzed, ranging from 3 to 10,000 residues. COC α DA is shown in cyan, SC in yellow, and NS in magenta. A detail of the 0–1,000 protein size range is shown in the upper left corner. Points represent individual entries, with lines showing the fitted curves for the data. Outliers were defined as ± 5 times the Standard Deviation for each approach.

Figure 6 shows the results for D2 when comparing the COC α DA (cyan), SC (yellow), and NS (magenta) approaches. It is possible to see that COC α DA performs better for all proteins, averaging approximately 6x faster times than NS and 2.5x faster times than SC. The SC approach performs better than NS in proteins below 5,000 residues, equal between 5,000 and 7,000 residues, and worse above 7,000 residues. However, since the vast majority of PDB entries fall within the smaller size range (approximately 97.2% of unique entries have 6,000 or fewer residues), where the performance gain of COC α DA and SC over NS is most pronounced, the high density of small proteins in the dataset significantly skews the overall average, leading to the reported 6x and 2.5x improvement.

Outliers were considered as entries that had a processing time ± 5 times the Standard Deviation for each approach, with less than 1% of entries removed. After outlier removal, it is possible to see that COC α DA has a consistent time vs. size distribution, while the other two approaches have more variation. This can be due to the tight and precise definition of cutoff distances for COC α DA, which speeds up a lot of the computation, while also limiting structural variations, like between globular and fibrillar proteins.

The protein size range of 0–1,000 residues is noteworthy, as shown in detail in the upper left corner of Figure 6. At this range, we can see the distribution pattern observed in D1, while also identifying several divergent entries in the NS approach. The divergent spike is composed exclusively of Nuclear Magnetic Resonance (NMR) resolved entries, which are usually deposited as several individual models of the same protein. Due to the nature of Biopython native parsing, all the models need to be parsed even if only the first one is of interest, unless the user creates specific functions for this purpose, thus deviating from the original implementation of the library. As these NMR entries are small, the parsing time of several NMR models outpaces the contact calculation time of the first one, leading to a spike in processing time. This does not occur in the COC α DA and SC approaches, as the customized parser handles only the selected model in the file (the default value is always the first model).

### 3.4 Empirical complexity analysis

Computing interatomic contacts is inherently a quadratic problem (O $\left(n^{2}\right)$ ) because it requires calculating the distance between every pair of atoms in a protein, such as in the AllAtoms approach. However, sophisticated data structures, such as k -d trees, can be employed to avoid calculating distances between atoms/residues that are too far apart. This approach theoretically reduces the computational space by pruning irrelevant comparisons, leading to practical reductions in computation time and typically logarithmic ( O(logn) , in the average of cases) or linear (O (n) , in the worst of cases) complexity (Bentley, 1975; Berg et al., 2008). However, these complexity values refer only to the operations of search, insertion, and deletion of elements in the trees. For the construction of a new tree—for example, for a new protein—the processing is substantially greater, corresponding to a log-linear complexity (O(nlogn)) in the best-case scenario, for balanced trees (Wald and Havran, 2006; Brown, 2015).

In the case of small entries, like most of the protein structures, the memory overhead associated with the allocation and creation of the tree usually does not outweigh the computational gains in search, insertion, and deletion operations. Thus, in addition to the high implementation complexity of k -d trees, the results may turn out to be worse than those obtained by ‘brute-force’ algorithms, provided that the latter are properly implemented. These gains are primarily concentrated in small proteins, which significantly contribute to the average observed speedup of 6x when comparing COC α DA to NS, even though the performance benefit becomes less pronounced on bigger proteins.

Our approach in COC α DA fits as an improvement of classical ‘brute-force’ algorithms (such as AllAtoms) for the calculation of interatomic contacts in proteins. This is because, theoretically, all atoms of the protein are checked at least once, but the precise definitions of cutoff distances for each residue pair significantly reduce processing time, without the additional cost of using complex data structures, as is the case with k -d trees.

In this study, we chose to evaluate the complexity of various algorithms empirically, by comparing standard methods commonly used in the structural bioinformatics community with the COC α DA method. These different methods were tested with inputs of increasing sizes (where n represents the number of residues, which have on average eight atoms each), and we analyzed the resulting fitted curves with real datasets.

The curve fittings of the three approaches against the second dataset demonstrate that both COC α DA ( $R^{2}$ quadratic = 0.99, Equation 3) and SC ( $R^{2}$ quadratic = 0.97, Equation 4) exhibit quadratic growth trends, while NS shows a linear growth trend ( $R^{2}$ linear = 0.97, Equation 5). This results demonstrate, experimentally, the nature of the contact identification functions, which are the most time-consuming operations. COC α DA and SC act basically as heavy improvements of ‘brute force’ algorithms, having a time complexity of $O\left(n^{2}\right)$ , while the NS contact identification function operates with a time complexity of O(n) , leading to its linear growth pattern.

$$f\left(n\right)=1.35\times 10^{-7}n^{2}+5.04\times 10^{-4}n-6.36\times 10^{-3};$$ (3)

$$g\left(n\right)=1.20\times 10^{-7}n^{2}+1.60\times 10^{-3}n-9.18\times 10^{-2};$$ (4)

$$h\left(n\right)=2.37\times 10^{-3}n+7.94\times 10^{-2},$$ (5)

where f(n) , g(n) , and h(n) are the best-fitted functions for COC α DA, SC, and NS, respectively, and n is the number of residues.

However, in the case of the NS approach, the memory overhead in tree construction is evident, especially for small inputs. It is possible to observe, in Figure 5, that for proteins of up to 200 residues even the most computationally expensive approach (AllAtoms) achieves lower processing times than NS. This can also be seen in the range that includes small entries in D2, although with less detail due to the massive number of points (Figure 6, detail).

The analysis of the coefficients from the obtained equations is another way to explain the poorer performance of NS, even though it grows linearly compared to the quadratic growth of the other approaches. Starting with the quadratic coefficients (a) , it can be noted that both are practically insignificant ( $1.35\times 10^{-7}$ for COC α DA and $1.20\times 10^{-7}$ for SC). This means that for small n values, as in D2, the quadratic growth is extremely slow, as can be seen from the slight curve in the data.

As for the linear coefficients (b) , there is a significant difference between COC α DA $\left(5.04\times 10^{-4}\right)$ and the other two approaches ( $1.60\times 10^{-3}$ for SC and $2.37\times 10^{-3}$ for NS), with NS showing the highest value. This difference, along with the low quadratic coefficients, explains how NS’s linear growth can be less efficient than the quadratic growth of the other approaches. Furthermore, as the SC and NS terms are close, between 6,000 and 7,000 residues the curves invert, representing the point where the quadratic growth of SC becomes more influential.

Finally, regarding the constant coefficients (c) , again there is a marked difference between the three approaches, with SC having the smallest value $\left(-9.18\times 10^{-3}\right)$ , followed by COC α DA $\left(-6.36\times 10^{-3}\right)$ , and NS $\left(7.94\times 10^{-2}\right)$ , the only approach with a positive c value. This result highlights the memory overhead associated with *k*-d tree construction, which causes even small inputs to have a baseline processing time higher than the actual contact calculation time.

As previously shown, the NS approach (using *k*-d trees) has a linear time complexity in the worst case. Meanwhile, COC α DA exhibits quadratic growth, yet shows lower processing times for all analyzed entries. By equating the two equations (Equation 3 for COC α DA; Equation 5 for NS), we find that COC α DA would only yield worse results for proteins with approximately 14,000 residues (Equation 6).

$$f\left(n\right)=h\left(n\right),\;\text{when }n\approx 14.000,$$ (6)

where f(n) e h(n) are the best-fitted functions to represent COC α DA and NS data, respectively, and n is the number of residues.

However, only slightly more than 2,000 entries in the PDB have a size greater than 14,000 residues, representing less than 1% of all protein structures. Furthermore, all of these entries correspond to protein complexes or repetitions of the same protein, as previously discussed. Therefore, for all practical purposes of contact detection in proteins, the COC α DA approach demonstrates the best temporal performance compared to other methodologies in the literature.

## 4 Conclusion

In a context where the influx of biological data is greater than ever, there is an increasing need for solutions that are efficient, robust, and scalable. In response to this demand, we developed COC α DA, a free, command-line tool designed to efficiently identify interatomic contacts in proteins at large scale. COC α DA employs a novel method for defining contact boundaries, based on the maximum distance between the alpha-carbons of amino acid pairs collected from all experimentally available proteins in the PDB.

Contact calculation between residues provides essential information on protein structure, function, stability, evolution, and molecular interactions. Although existing algorithms perform well for individual structures, their computational cost can become limiting in large-scale applications, such as analysis of structural databases like the PDB and AFDB, and molecular dynamics simulations where contacts must be recalculated for thousands of frames. More efficient methods, like the one we present here, enable faster processing and broader analyses across large datasets.

By leveraging structural and physicochemical knowledge of amino acids, we derived optimal main-chain alpha-carbon cutoff values for each amino acid pair, which significantly reduces the computational cost of detecting interatomic contacts. Advanced data structures such as k -d trees are efficient for large entries but introduce unnecessary overhead for the smaller proteins that dominate the PDB. Instead, COC α DA employs a structure-based approach that prunes the search space based on C α distances, which undergo fewer conformational changes than side-chain atoms.

This approach led to a stable and efficient method tailored to real-world structural datasets. This strategic simplicity not only outperforms more complex alternatives in practice but also simplifies implementation by requiring neither external libraries nor advanced programming skills. Its scalable performance makes COC α DA suitable for a wide range of structural bioinformatics applications, including macromolecular interaction modeling, functional site prediction, high-throughput structural analysis, and studies of protein evolution.

The current version of COC α DA generates a ‘.csv’ output file containing comprehensive information for each detected contact, including chain name, residue name and number, atom name, distance between the atom pairs, and contact type. Because the tool identifies contacts across all residues, the results can be classified as either intra-chain or inter-chain contacts, the latter being particularly valuable for analyses of protein-protein or protein-ligand interfaces. COC α DA is implemented in Python, and the full source code is publicly available at https://github.com/LBS-UFMG/COCaDA.

## Data Availability

The datasets presented in this study can be found in online repositories. This data can be found here: https://github.com/lbs-ufmg/cocada_supplementary.

## Footnotes

1 Available at https://www.rcsb.org/stats/explore/polymer_entity_type. Accessed 23 August 2024.

2 Available at https://www.rcsb.org/stats/growth/growth-protein. Accessed 23 August 2024.

3 Available at https://github.com/PDBeurope/arpeggio/.
