# Supplementary material for: COC α DA - a fast and scalable algorithm for interatomic contact detection in proteins using C α distance matrices
Source: Front Bioinform. 2025 Sep 1;5:1630078. doi: 10.3389/fbinf.2025.1630078 (PMC12433948; doi:10.3389/fbinf.2025.1630078)
Supplement: Supplementary file 1 [file DataSheet1.pdf]

Supplementary Material

All other supplementary material, including the NS implementation source code, the 896 '.pdb' files used in the first dataset (D1), and the list of 217,454 PDB IDs used to generate the maximum distance matrix, can be accessed on `https://github.com/LBS-UFMG/COCaDA_supplementary`.

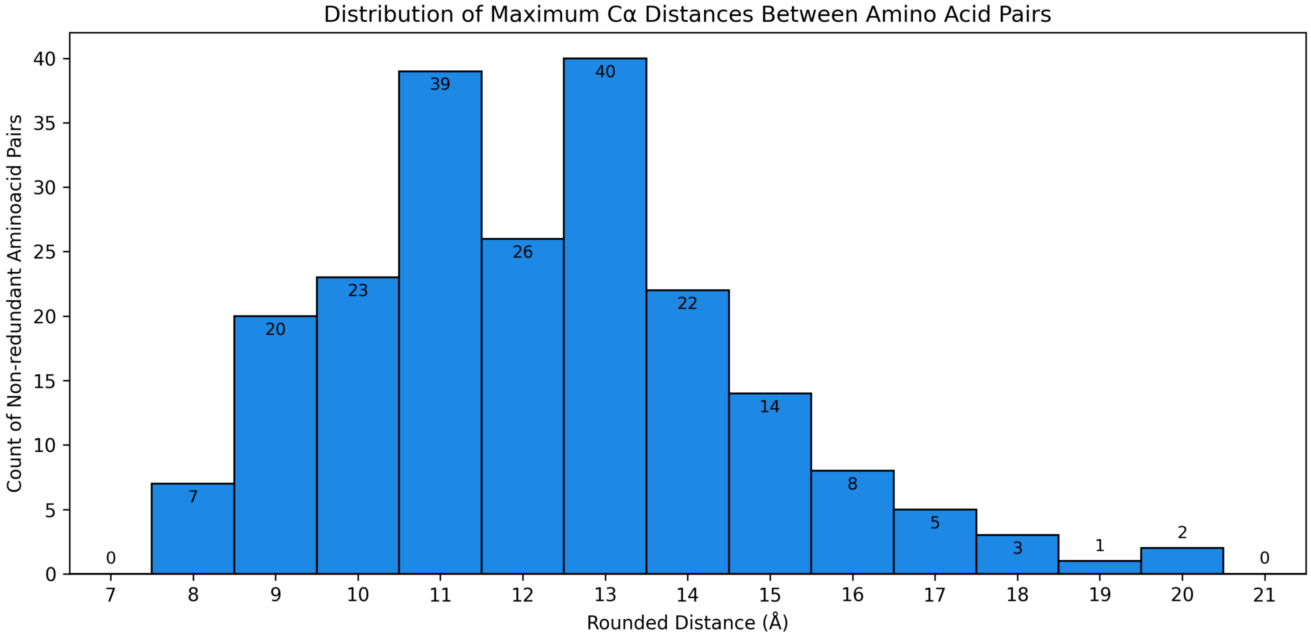

Figure S1: **Histogram plot of the distribution of the maximum Cα distances between all amino acid pairs.** The x axis represents the maximum rounded Cα distance, and the y axis the count of non-redundant pairs that fall in each bin.

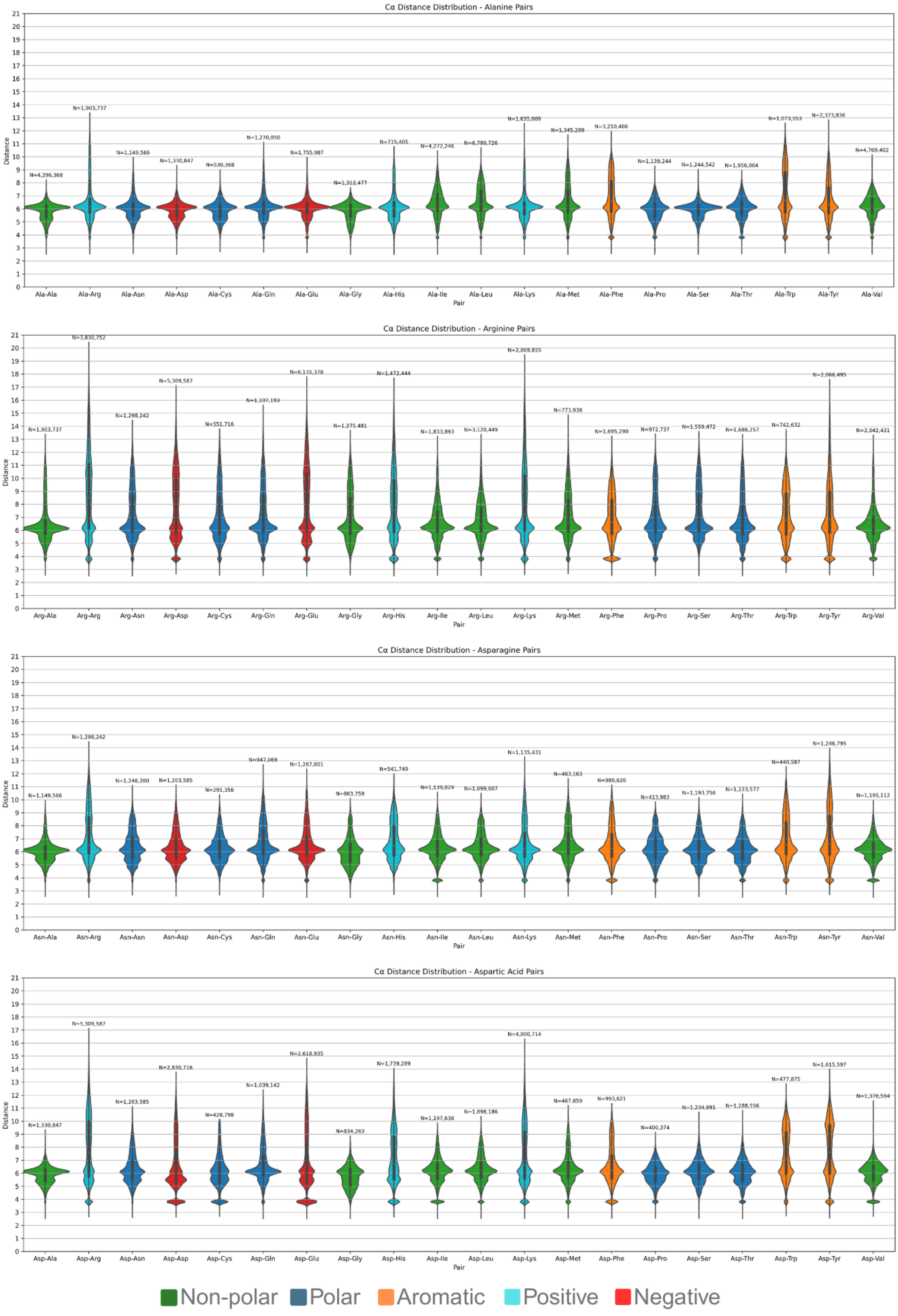

Figure S2. **Full Cα distance distribution across Dataset 2 for Alanine, Arginine, Asparagine and Aspartic Acid pairs.** Full caption on page 7.

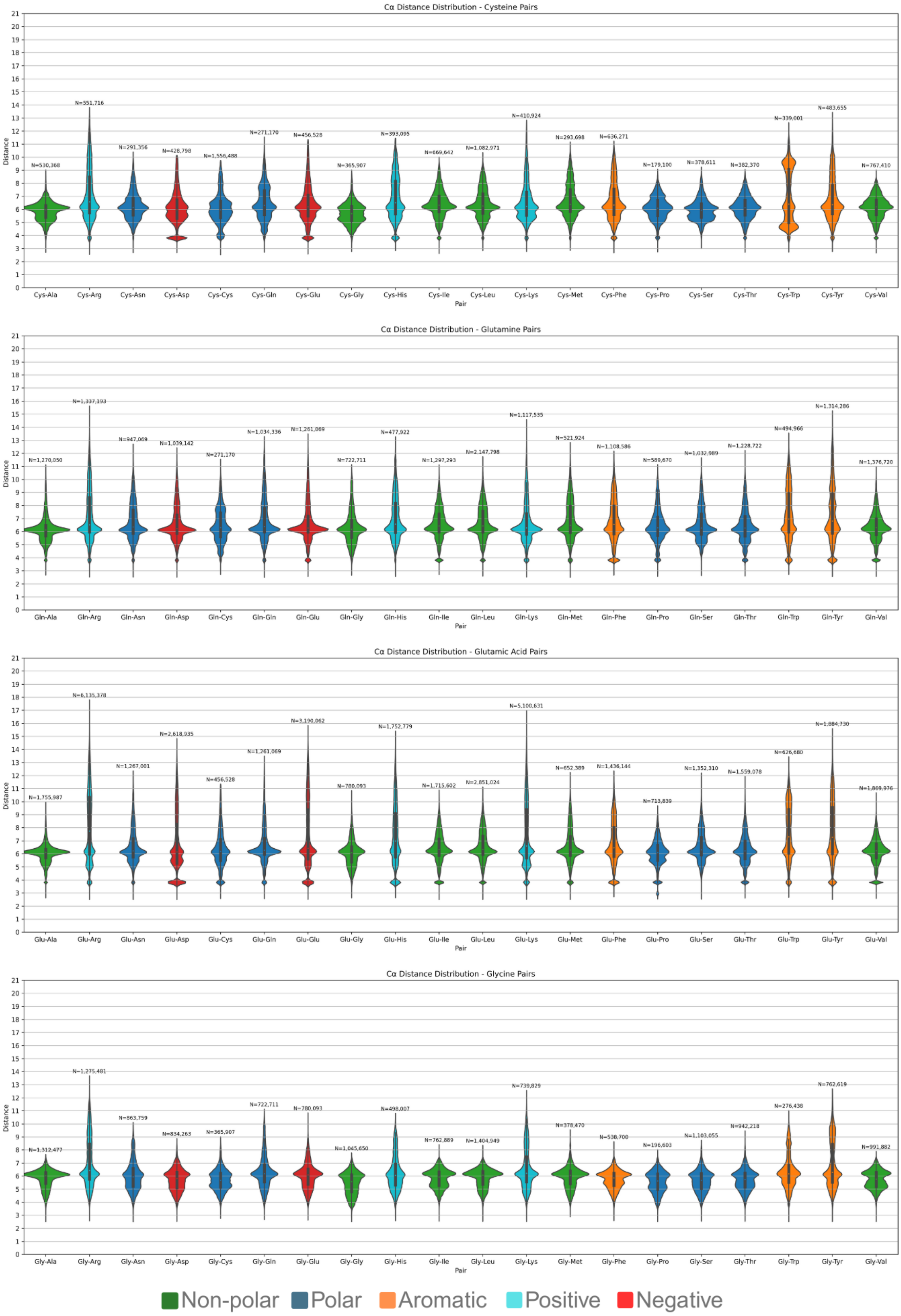

Figure S2. **Full Cα distance distribution across Dataset 2 for Cysteine, Glutamine, Glutamic Acid and Glycine pairs.** Full caption on page 7.

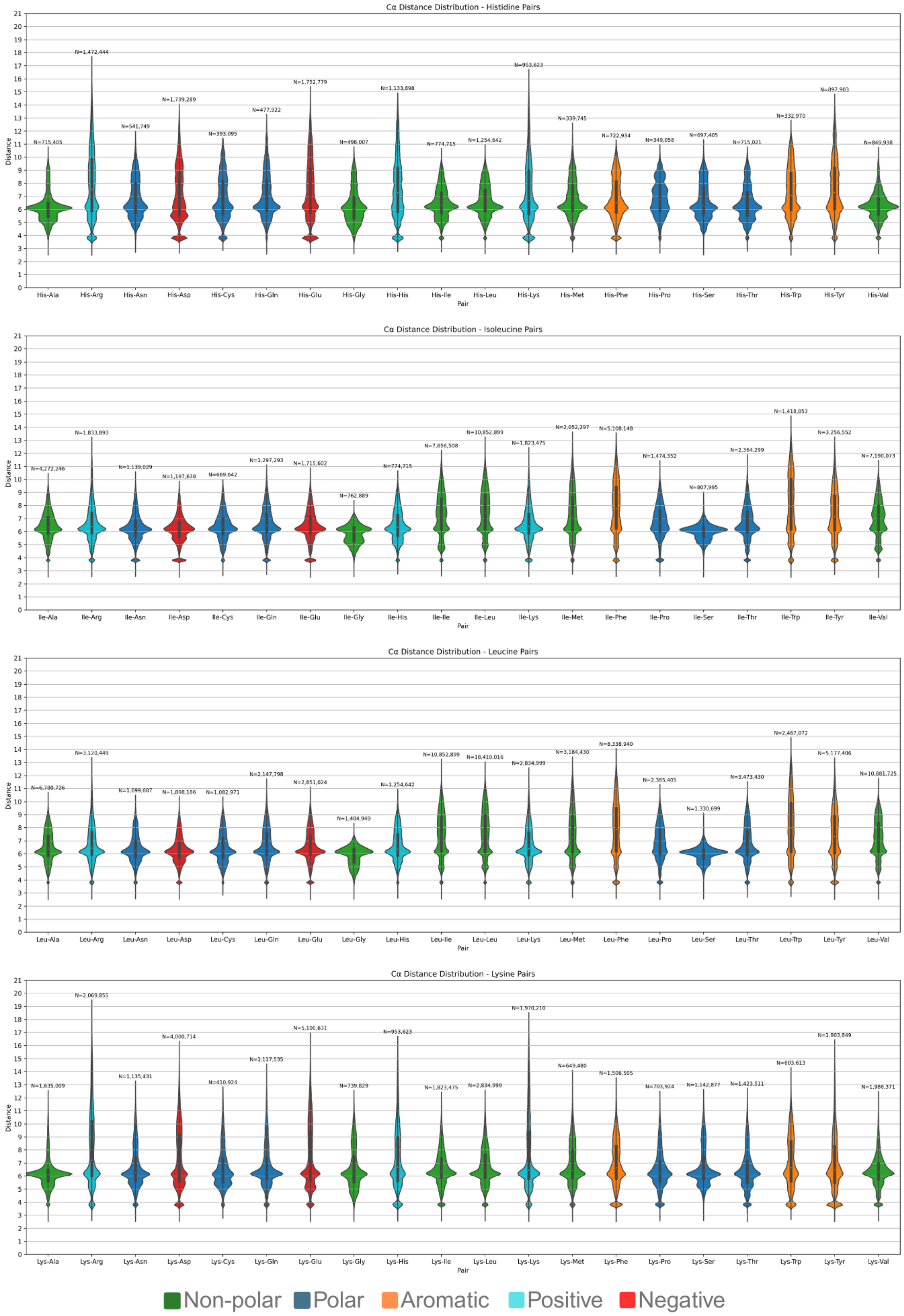

Figure S2. **Full Cα distance distribution across Dataset 2 for Histidine, Isoleucine, Leucine and Lysine pairs.** Full caption on page 7.

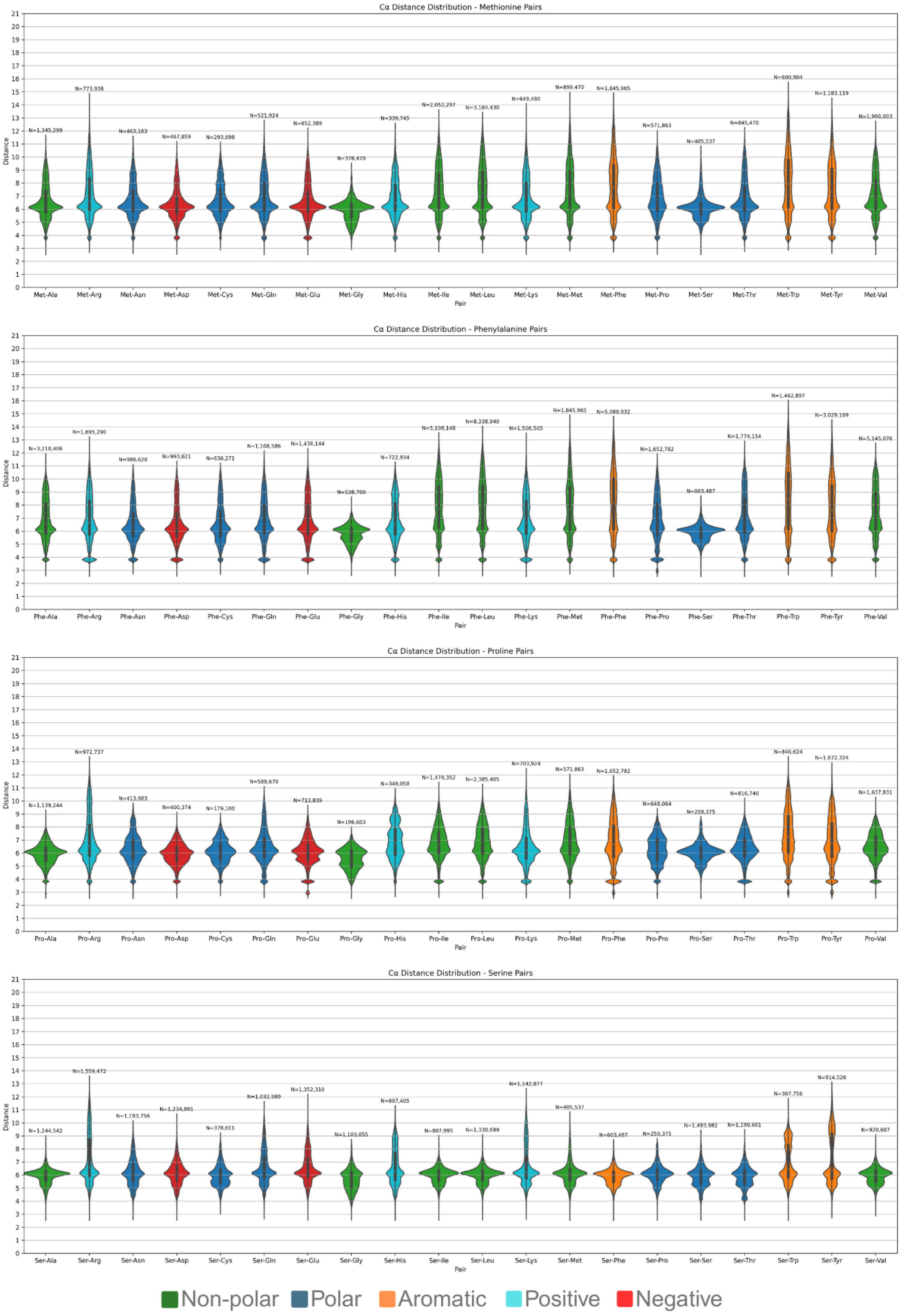

Figure S2. **Full Cα distance distribution across Dataset 2 for Methionine, Phenylalanine, Proline and Serine pairs.** Full caption on page 7.

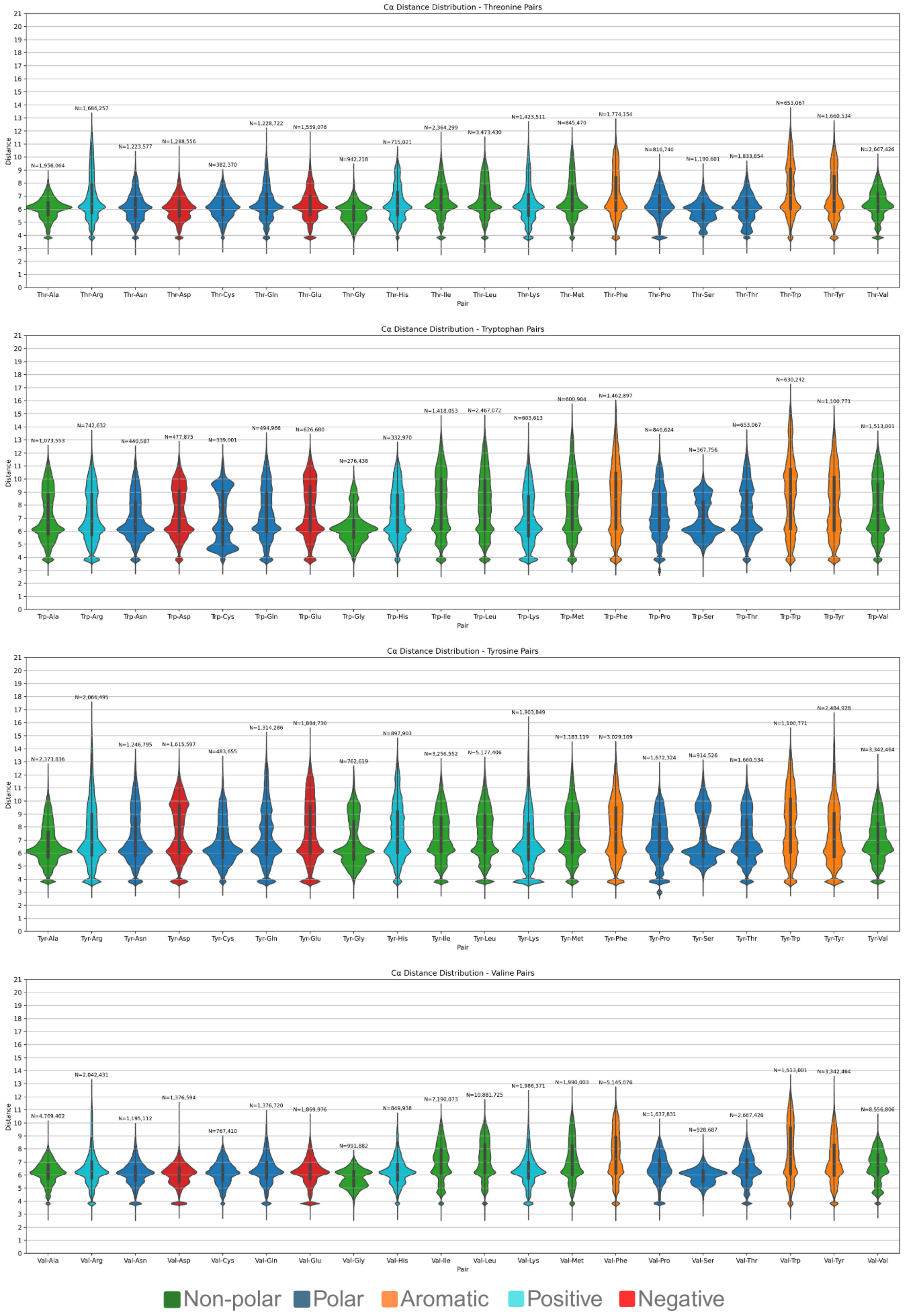

Figure S2. **Full Cα distance distribution across Dataset 2 for Threonine, Tryptophan, Tyrosine and Valine pairs.** Full caption on page 7.

Figure S2: **Cα distance distribution for all amino acid residue pairs in Dataset 2.** Each graph shows 20 violin plots representing the distributions of Cα-Cα distances of an amino acid residue. Each individual plot corresponds to a specific pair on the x-axis, with distances plotted in angstroms. Residue types are color-coded based on their physicochemical properties: green – non-polar; blue – polar; orange – aromatic; cyan – positively charged; red – negatively charged. The number shown above each violin plot indicates the total number of interacting residue pairs considered for that specific combination. Only residue pairs involved in some form of interaction were included in the analysis, ensuring the distributions reflect biologically relevant contacts. The y-axis limit was set according to the maximum observed Cα distance, which occurred for the Arg–Arg pair, to maintain consistency across plots. Redundant pairs (e.g., Ala–Gly and Gly–Ala) were both included to reflect the full range of interaction contexts.

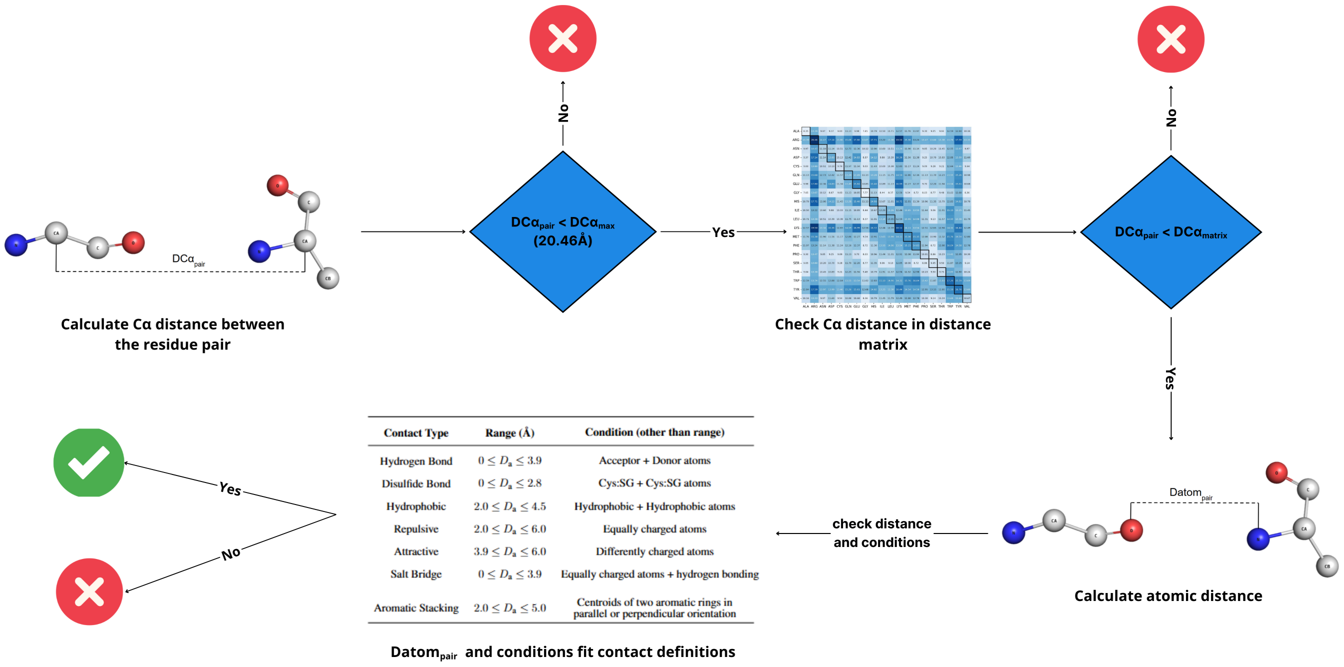

Figure S3: **Schematic illustration of the pruning process.** To improve computational efficiency, residue pairs are filtered in three steps. First, a coarse filter eliminates pairs with Cα–Cα distances (DCα_{pair}) exceeding a global maximum cutoff of 20.46Å (DCα_{max}), allowing early removal of clearly non-interacting pairs. Second, remaining pairs are then subjected to a more stringent, pair-specific cutoff comparison using the distance matrix (DCα_{pair} < DCα_{matrix}). Finally, pairs passing both Cα-based criteria undergo atomic-level evaluation, where the presence of a contact is determined by assessing atom–atom distances (Datom$_{pair}$) and specific interaction criteria as defined in Table 2. Only pairs meeting all criteria are classified as contacts.

Table S1. **Binary classification of heavy atoms, according to their characteristics.** For each amino acid residue, all their heavy atoms were classified in a binary manner, according to the following characteristics (hydrophobic, aromatic, positive, negative, donor, acceptor). Atom names follow the PDB nomenclature. Data in Python dictionary form is available at `https://github.com/LBS-UFMG/COCaDA/blob/main/src/conditions.py`.

Residue	Atom	Hydrophobic	Aromatic	Positive	Negative	Donor	Acceptor
Alanine	N	0	0	0	0	1	0
Alanine	CA	0	0	0	0	0	0
Alanine	C	0	0	0	0	0	0
Alanine	O	0	0	0	0	0	1
Alanine	CB	1	0	0	0	0	0
Arginine	N	0	0	0	0	1	0
Arginine	CA	0	0	0	0	0	0
Arginine	C	0	0	0	0	0	0
Arginine	O	0	0	0	0	0	1
Arginine	CB	1	0	0	0	0	0
Arginine	CG	1	0	0	0	0	0
Arginine	CD	0	0	0	0	0	0
Arginine	NE	0	0	1	0	1	0
Arginine	CZ	0	0	1	0	0	0
Arginine	NH1	0	0	1	0	1	0
Arginine	NH2	0	0	1	0	1	0
Asparagine	N	0	0	0	0	1	0
Asparagine	CA	0	0	0	0	0	0
Asparagine	C	0	0	0	0	0	0
Asparagine	O	0	0	0	0	0	1
Asparagine	CB	1	0	0	0	0	0
Asparagine	CG	0	0	0	0	0	0
Asparagine	OD1	0	0	0	0	0	1
Asparagine	ND2	0	0	0	0	1	0
Aspartate	N	0	0	0	0	1	0
Aspartate	CA	0	0	0	0	0	0
Aspartate	C	0	0	0	0	0	0
Aspartate	O	0	0	0	0	0	1
Aspartate	CB	1	0	0	0	0	0
Aspartate	CG	0	0	0	0	0	0
Aspartate	OD1	0	0	0	1	0	1
Aspartate	OD2	0	0	0	1	0	1
Cysteine	N	0	0	0	0	1	0
Cysteine	CA	0	0	0	0	0	0
Cysteine	C	0	0	0	0	0	0
Cysteine	O	0	0	0	0	0	1
Cysteine	CB	1	0	0	0	0	0

Continued on next page

Table S1 – continued from previous page

Residue	Atom	Hydrophobic	Aromatic	Positive	Negative	Donor	Acceptor
Cysteine	SG	0	0	0	0	1	1
Glutamine	N	0	0	0	0	1	0
Glutamine	CA	0	0	0	0	0	0
Glutamine	C	0	0	0	0	0	0
Glutamine	O	0	0	0	0	0	1
Glutamine	CB	1	0	0	0	0	0
Glutamine	CG	1	0	0	0	0	0
Glutamine	CD	0	0	0	0	0	0
Glutamine	OE1	0	0	0	0	0	1
Glutamine	NE2	0	0	0	0	1	0
Glutamate	N	0	0	0	0	1	0
Glutamate	CA	0	0	0	0	0	0
Glutamate	C	0	0	0	0	0	0
Glutamate	O	0	0	0	0	0	1
Glutamate	CB	1	0	0	0	0	0
Glutamate	CG	1	0	0	0	0	0
Glutamate	CD	0	0	0	0	0	0
Glutamate	OE1	0	0	0	1	0	1
Glutamate	OE2	0	0	0	1	0	1
Glycine	N	0	0	0	0	1	0
Glycine	CA	0	0	0	0	0	0
Glycine	C	0	0	0	0	0	0
Glycine	O	0	0	0	0	0	1
Histidine	N	0	0	0	0	1	0
Histidine	CA	0	0	0	0	0	0
Histidine	C	0	0	0	0	0	0
Histidine	O	0	0	0	0	0	1
Histidine	CB	1	0	0	0	0	0
Histidine	CG	0	1	0	0	0	0
Histidine	ND1	0	1	1	0	1	1
Histidine	CD2	0	1	0	0	0	0
Histidine	CE1	0	1	0	0	0	0
Histidine	NE2	0	1	1	0	1	1
Isoleucine	N	0	0	0	0	1	0
Isoleucine	CA	0	0	0	0	0	0
Isoleucine	C	0	0	0	0	0	0
Isoleucine	O	0	0	0	0	0	1
Isoleucine	CB	1	0	0	0	0	0
Isoleucine	CG1	1	0	0	0	0	0
Isoleucine	CG2	1	0	0	0	0	0
Isoleucine	CD1	1	0	0	0	0	0
Leucine	N	0	0	0	0	1	0

Continued on next page

Table S1 – continued from previous page

Residue	Atom	Hydrophobic	Aromatic	Positive	Negative	Donor	Acceptor
Leucine	CA	0	0	0	0	0	0
Leucine	C	0	0	0	0	0	0
Leucine	O	0	0	0	0	0	1
Leucine	CB	1	0	0	0	0	0
Leucine	CG	1	0	0	0	0	0
Leucine	CD1	1	0	0	0	0	0
Leucine	CD2	1	0	0	0	0	0
Lysine	N	0	0	0	0	1	0
Lysine	CA	0	0	0	0	0	0
Lysine	C	0	0	0	0	0	0
Lysine	O	0	0	0	0	0	1
Lysine	CB	1	0	0	0	0	0
Lysine	CG	1	0	0	0	0	0
Lysine	CD	1	0	0	0	0	0
Lysine	CE	0	0	0	0	0	0
Lysine	NZ	0	0	1	0	1	0
Methionine	N	0	0	0	0	1	0
Methionine	CA	0	0	0	0	0	0
Methionine	C	0	0	0	0	0	0
Methionine	O	0	0	0	0	0	1
Methionine	CB	1	0	0	0	0	0
Methionine	CG	1	0	0	0	0	0
Methionine	SD	0	0	0	0	0	1
Methionine	CE	1	0	0	0	0	0
Phenylalanine	N	0	0	0	0	1	0
Phenylalanine	CA	0	0	0	0	0	0
Phenylalanine	C	0	0	0	0	0	0
Phenylalanine	O	0	0	0	0	0	1
Phenylalanine	CB	1	0	0	0	0	0
Phenylalanine	CG	1	1	0	0	0	0
Phenylalanine	CD1	1	1	0	0	0	0
Phenylalanine	CD2	1	1	0	0	0	0
Phenylalanine	CE1	1	1	0	0	0	0
Phenylalanine	CE2	1	1	0	0	0	0
Phenylalanine	CZ	1	1	0	0	0	0
Proline	N	0	0	0	0	0	0
Proline	CA	0	0	0	0	0	0
Proline	C	0	0	0	0	0	0
Proline	O	0	0	0	0	0	1
Proline	CB	1	0	0	0	0	0
Proline	CG	1	0	0	0	0	0
Proline	CD	0	0	0	0	0	0

Continued on next page

Table S1 – continued from previous page

Residue	Atom	Hydrophobic	Aromatic	Positive	Negative	Donor	Acceptor
Serine	N	0	0	0	0	1	0
Serine	CA	0	0	0	0	0	0
Serine	C	0	0	0	0	0	0
Serine	O	0	0	0	0	0	1
Serine	CB	0	0	0	0	0	0
Serine	OG	0	0	0	0	1	1
Threonine	N	0	0	0	0	1	0
Threonine	CA	0	0	0	0	0	0
Threonine	C	0	0	0	0	0	0
Threonine	O	0	0	0	0	0	1
Threonine	CB	0	0	0	0	0	0
Threonine	OG1	0	0	0	0	1	1
Threonine	CG2	1	0	0	0	0	0
Tryptophan	N	0	0	0	0	1	0
Tryptophan	CA	0	0	0	0	0	0
Tryptophan	C	0	0	0	0	0	0
Tryptophan	O	0	0	0	0	0	1
Tryptophan	CB	1	0	0	0	0	0
Tryptophan	CG	1	1	0	0	0	0
Tryptophan	CD1	0	1	0	0	0	0
Tryptophan	CD2	1	1	0	0	0	0
Tryptophan	NE1	0	1	0	0	1	0
Tryptophan	CE2	0	1	0	0	0	0
Tryptophan	CE3	1	1	0	0	0	0
Tryptophan	CZ2	1	1	0	0	0	0
Tryptophan	CZ3	1	1	0	0	0	0
Tryptophan	CH2	1	1	0	0	0	0
Tyrosine	N	0	0	0	0	1	0
Tyrosine	CA	0	0	0	0	0	0
Tyrosine	C	0	0	0	0	0	0
Tyrosine	O	0	0	0	0	0	1
Tyrosine	CB	1	0	0	0	0	0
Tyrosine	CG	1	1	0	0	0	0
Tyrosine	CD1	1	1	0	0	0	0
Tyrosine	CD2	1	1	0	0	0	0
Tyrosine	CE1	1	1	0	0	0	0
Tyrosine	CE2	1	1	0	0	0	0
Tyrosine	CZ	0	1	0	0	0	0
Tyrosine	OH	0	0	0	0	1	1
Valine	N	0	0	0	0	1	0
Valine	CA	0	0	0	0	0	0
Valine	C	0	0	0	0	0	0

Continued on next page

Table S1 – continued from previous page

Residue	Atom	Hydrophobic	Aromatic	Positive	Negative	Donor	Acceptor
Valine	O	0	0	0	0	0	1
Valine	CB	1	0	0	0	0	0
Valine	CG1	1	0	0	0	0	0
Valine	CG2	1	0	0	0	0	0

Table S2. Comparison of contacts identified in dataset D1 using the AllAtoms, SC, NS and CO$C\alpha$DA approaches. Each row shows the number of contacts of a specific type detected, as well as the total number of contacts in D1.

**Contact Type**	**AllAtoms**	**SC**	**NS**	**CO$C\alpha$DA**
Hydrophobic	801,034	801,034	801,034	801,034
Hydrogen Bond	333,465	333,465	333,465	333,465
Attractive	98,500	98,500	98,500	98,500
Repulsive	58,813	58,813	58,813	58,813
Salt Bridge	44,293	44,293	44,293	44,293
Aromatic Stacking	3,426	3,426	3,426	3,426
Disulfide Bond	54	54	54	54
**Total**	**1,339,585**	**1,339,585**	**1,339,585**	**1,339,585**

Table S3. **Maximum Cα distance of all pairs of residues.** Each column contain half of the pairs, in ascending order. All values were added 0.01Å to handle small rounding errors. Data in Python dictionary form is available at `https://github.com/LBS-UFMG/COCaDA/blob/main/src/distances.py`.

Residue Pair	Distance	Residue Pair	Distance
Ala - Gly	7.66	Phe - Gln	12.17
Gly - Gly	7.78	Glu - Ser	12.21
Gly - Pro	8.04	Gln - Thr	12.24
Ala - Ala	8.36	Ile - Ile	12.24
Gly - Val	8.37	Glu - Met	12.28
Gly - Leu	8.38	Met - Thr	12.33
Gly - Ile	8.45	Glu - Asn	12.37
Phe - Gly	8.73	Glu - Phe	12.38
Phe - Ser	8.73	Asp - Gln	12.43
Gly - Ser	8.78	Ile - Lys	12.45
Pro - Ser	8.87	Lys - Val	12.5
Asp - Gly	8.88	Lys - Pro	12.52
Ala - Cys	9.04	Asn - Trp	12.56
Cys - Gly	9.04	Gly - Lys	12.57
Ala - Thr	9.05	Ala - Lys	12.58
Ala - Ser	9.06	Ala - Trp	12.6
Ile - Ser	9.07	Lys - Leu	12.6
Cys - Pro	9.1	Cys - Trp	12.65
Leu - Ser	9.14	Lys - Ser	12.66
Ser - Val	9.15	Gly - Tyr	12.69
Cys - Thr	9.22	Asn - Gln	12.74
Asp - Pro	9.26	Phe - Val	12.79
Cys - Ser	9.29	Met - Val	12.81
Ala - Pro	9.31	Thr - Tyr	12.81
Ala - Asp	9.38	Cys - Lys	12.84
Ser - Ser	9.46	His - Trp	12.84
Gly - Thr	9.49	Ala - Tyr	12.85
Ser - Thr	9.51	Met - Gln	12.87
Cys - Val	9.55	Asp - Trp	12.89
Gly - Met	9.55	His - Met	12.92
Glu - Pro	9.71	Lys - Thr	12.95
Cys - Cys	9.75	Pro - Tyr	12.96
Thr - Thr	9.76	Phe - Thr	12.99
Asn - Pro	9.86	Leu - Leu	13.06
Asp - Ile	9.89	Ser - Tyr	13.16
Ala - Asn	9.98	Ile - Arg	13.23
Asn - Val	9.98	Phe - Arg	13.25

Continued on next page

Table S3 – continued from previous page

Residue Pair	Distance	Residue Pair	Distance
Ala - Glu	9.99	Ile - Tyr	13.26
Pro - Pro	10.02	Ile - Leu	13.27
Cys - Ile	10.04	His - Gln	13.29
Gly - Asn	10.13	Gln - Gln	13.3
Cys - Asp	10.14	Arg - Val	13.34
Ala - Val	10.17	Lys - Asn	13.35
Asn - Ser	10.21	Arg - Thr	13.39
Pro - Thr	10.24	Leu - Arg	13.39
Thr - Val	10.25	Leu - Tyr	13.39
Pro - Val	10.31	Ala - Arg	13.4
Cys - Leu	10.39	Pro - Trp	13.43
Asp - Leu	10.4	Cys - Tyr	13.47
Asn - Thr	10.46	Pro - Arg	13.48
Ala - Ile	10.51	Glu - Gln	13.5
Leu - Asn	10.52	Glu - Trp	13.55
Cys - Asn	10.52	Phe - Lys	13.55
Ile - Asn	10.61	Gln - Trp	13.56
Val - Val	10.68	Phe - Ile	13.56
His - Ile	10.68	Val - Tyr	13.61
Glu - Val	10.69	Arg - Ser	13.61
Asp - Ser	10.71	Ile - Met	13.66
Ala - Leu	10.72	Gly - Arg	13.68
His - Val	10.8	Val - Trp	13.7
Ala - His	10.8	Arg - Trp	13.75
His - Thr	10.8	Cys - Arg	13.81
Asp - Thr	10.84	Thr - Trp	13.84
Glu - Gly	10.86	Asp - Asp	13.86
Glu - Ile	10.9	Asn - Tyr	13.98
Met - Ser	10.91	Leu - Met	14.0
His - Pro	10.97	Asp - Tyr	14.0
Gln - Val	10.99	Phe - Leu	14.07
His - Leu	11.02	Lys - Met	14.12
Gly - Trp	11.03	Asp - His	14.13
Asn - Asn	11.11	Lys - Trp	14.33
Gly - His	11.14	Asn - Arg	14.48
Glu - Leu	11.14	Met - Tyr	14.55
Pro - Gln	11.14	Phe - Tyr	14.59
Ala - Gln	11.14	Lys - Gln	14.6
Gly - Gln	11.14	His - Tyr	14.83
Asp - Asn	11.15	Asp - Glu	14.84

Continued on next page

Table S3 – continued from previous page

Residue Pair	Distance	Residue Pair	Distance
Phe - Asn	11.15	Phe - Phe	14.84
Ile - Gln	11.16	Leu - Trp	14.91
Cys - Met	11.18	His - His	14.94
Cys - Phe	11.25	Met - Met	15.01
Phe - His	11.31	Ile - Trp	15.11
Leu - Pro	11.32	Phe - Met	15.16
Cys - Glu	11.35	Gln - Tyr	15.27
His - Ser	11.36	Met - Arg	15.39
Asp - Phe	11.39	Glu - His	15.41
Cys - His	11.44	Trp - Tyr	15.6
Ile - Pro	11.45	Glu - Tyr	15.62
Ile - Val	11.46	Gln - Arg	15.69
Asp - Met	11.57	Met - Trp	15.77
Leu - Thr	11.58	Glu - Glu	15.85
Cys - Gln	11.58	Phe - Trp	16.05
Asp - Val	11.61	Asp - Lys	16.37
Gln - Ser	11.71	Lys - Tyr	16.45
Leu - Gln	11.76	His - Lys	16.73
Ala - Met	11.77	Tyr - Tyr	16.77
Leu - Val	11.8	Glu - Lys	17.0
Ser - Trp	11.88	Asp - Arg	17.15
Met - Asn	11.91	Trp - Trp	17.27
Ile - Thr	11.92	Arg - Tyr	17.6
Glu - Thr	11.95	His - Arg	17.72
Phe - Pro	11.95	Glu - Arg	17.81
Ala - Phe	11.98	Lys - Lys	18.53
His - Asn	12.01	Lys - Arg	19.51
Met - Pro	12.09	Arg - Arg	20.47
